# An Improved Distance Matrix Computation Algorithm for Multicore Clusters

**DOI:** 10.1155/2014/406178

**Published:** 2014-06-12

**Authors:** Mohammed W. Al-Neama, Naglaa M. Reda, Fayed F. M. Ghaleb

**Affiliations:** ^1^Department of Mathematics, Faculty of Science, Al-Azhar University, Cairo, Egypt; ^2^Education College for Girls, Mosul University, Mosul, Iraq; ^3^Department of Mathematics, Faculty of Science, Ain Shams University, Cairo, Egypt

## Abstract

Distance matrix has diverse usage in different research areas. Its computation is typically an essential task in most bioinformatics applications, especially in multiple sequence alignment. The gigantic explosion of biological sequence databases leads to an urgent need for accelerating these computations. *DistVect* algorithm was introduced in the paper of Al-Neama et al. (in press) to present a recent approach for vectorizing distance matrix computing. It showed an efficient performance in both sequential and parallel computing. However, the multicore cluster systems, which are available now, with their scalability and performance/cost ratio, meet the need for more powerful and efficient performance. This paper proposes *DistVect1* as highly efficient parallel vectorized algorithm with high performance for computing distance matrix, addressed to multicore clusters. It reformulates *DistVect1* vectorized algorithm in terms of clusters primitives. It deduces an efficient approach of partitioning and scheduling computations, convenient to this type of architecture. Implementations employ potential of both MPI and OpenMP libraries. Experimental results show that the proposed method performs improvement of around 3-fold speedup upon SSE2. Further it also achieves speedups more than 9 orders of magnitude compared to the publicly available parallel implementation utilized in ClustalW-MPI.

## 1. Introduction

Distance matrix (DM) refers to a two-dimensional array containing the pairwise distances of a set of elements. DM has a broad range of usage in various scientific research fields. It is used intensively in data clustering [1], pattern recognition [2], image analysis [3], information retrieval [4], and bioinformatics. In bioinformatics, it is mainly used in constructing the so-called phylogenetic tree, which is a diagram that depicts the lines of evolutionary descent of different species, organisms, or genes from a common ancestor [5].

The explosive growth of genomes makes the ability to align a huge number of long sequences become more essential. For example, the Ribosomal Database Project Release 10 [6] consists of more than million sequences. This leads to the massive number of distance calculations. As, for just aligning 100,000 sequences, approximately 5 billion distances need to be computed to construct a complete DM. Even if the sequences are short and pairwise distance calculations can be done relatively quickly, say at a rate of 5,000^−1^ sec., their alignment still requires almost 12 days of CPU time. Another difficulty is how to store the DM elements, as it will take up to 40 GB of memory. This leads to the need of new approaches to accelerate the distance calculations and to handle storage efficiently.

On the other hand, the rapid development of high performance computing (HPC) hardware provides high-performance cost ratio computational capability. The high precisions of multicore computers, clusters, and grids have become increasingly available and more powerful nowadays [7]. Therefore, it is of interest to use high-performance technologies to unlock the potential of such systems. Meanwhile, parallel programming libraries such as OpenMP and MPI made it possible for programmers to take advantage of the great computational capability of multicore and clusters for general purpose usage.

Many attempts have been produced to efficiently compute the distance matrix. As seen in the next section, the ones that used GPUs are really fast but the length of sequences is limited. Other methods that can handle long sequences and produce accurate alignment are relatively slow. Our motivation is to provide an efficient method that combines the speed and the ability to align long sequences.

This paper is an extension to our work in [8]. An improved version of* DistVect* algorithm is proposed. The computations are redistributed efficiently to manage the load imbalance aiming at enhancing the overall performance. The algorithm has been upgraded to the hybrid model so as to reap the maximum benefits of fine- and coarse-grained models. The proposed method is devoted to multicore clusters due to its popularity nowadays and is for achieving higher processing speed.

The main contributions of this paper are:

- designing a highly parallel extended* DistVect* algorithm for distance matrix computation on multicore clusters, called* DistVect1*, to align huge sequences fast,
- implementing the proposed* DistVect1* algorithm using C++ with MPI and OpenMP on Bibliotheca Alexandrina platform,
- carrying out comprehensive experiments using a wide variety of real dataset sizes and showing that our developed program outperforms both ClustalW-MPI and SSE2 in terms of execution time,
- investigating the impact of increasing both the number and the length of sequences on the speedup and demonstrating that* DistVect1* yields significant speedup when the length of genomes increases.

The rest of this paper is organized as follows.  Section 2 summarizes briefly the fundamental methods and algorithms concerning the distance matrix.  Section 3 explains the* DistVect* algorithm.  Section 4 discusses the improved* DistVect1*.  Section 5 describes the implementation procedure briefly and presents results with a detailed analysis. Finally, Section 6 concludes the paper and suggests future work.

## 2. Related Work

Distance matrix computation is considered as the substantial stage of most multiple sequence alignment tools. To align a dataset of size *N* × *L*, where *N* is the number of sequences and *L* is their average length, the computation of the DM elements requires ⌈*N*(*N* − 1)/2⌉ pairwise comparisons. Each comparison uses a matrix of size (*L* + 1)×(*L* + 1) to obtain the distance. These computations can become prohibitive when *N* and *L* are very large (i.e., in the tens of thousands). There are few multiple alignment programs that handle datasets of this size, with acceptable accuracy, such as MAFFT [9], DIALIGN [10], and Clustal [11]. Most accurate methods could only routinely handle hundreds or few thousands of sequences, like MUSCLE [12], Probcoms [13], and T-Coffee [14].

Promising solutions have been found for parallelizing DM calculations. Various parallel algorithms were presented to overcome speed/space obstacles for different HPC systems such as multiprocessor machines and workstation clusters. One category focuses on parallelizing the operations on smaller data components. Typical implementations of this approach using multithreading are in [15, 16]. The others concentrate on distributing each independent pair of sequences on different processors. The most popular parallel method that uses this approach is ClustalW-MPI [17]. It is targeted for workstation clusters with distributed memory architecture. Its main contribution was providing an efficient distributed memory implementation of ClustalW that can be run on a wide range of distributed memory PC clusters and parallel multicomputers.

Wirawan et al. exploit in [18] the use of an intertask approach, with SIMD model. They take advantage of the fact that all elements in the same minor diagonal can be computed independently in parallel. They used common Intel processors with the SSE2 instruction set, supporting 16-bit elements, and produced a software tool called SSE2, which was mainly written in C with p-thread API. This approach has been exploited in some recent methods [15, 19–21] where parallelism occurs within a single pair of sequences, to avoid data dependencies within the alignment matrix.

GPU has been used in [22] to accelerate sequence alignment. It has reformulated dynamic programming-based alignment algorithms as streaming algorithms in terms of computer graphics primitives. Experimental results show that the GPU-based approach allows speedups of over one order of magnitude with respect to optimized CPU implementations. Nevertheless, this is not severe since 99.8 percent of the sequences in the database are of length <4,096. Furthermore, it is reasonable to expect that the allowed texture buffer sizes will increase in next-generation graphics hardware.

Also, CUDASW++ [20] parallelizes Smith-Waterman algorithm for CUDA GPU that computes the similarity scores of a query sequence paired with each sequence in a database. Performance analysis shows substantial improvement to the overall performance on the order of three to four giga-cell updates per second. The single-GPU version achieves an average performance of 9.509 GCUPS with a lowest performance of 9.039 GCUPS and a highest performance of 9.660 GCUPS, and the dual-GPU version achieves an average performance of 14.484 GCUPS with a lowest performance of 10.660 GCUPS and a highest performance of 16.087 GCUPS. But it supports query sequences of length up to 59 K and for query sequences ranging in length from 144 to 5,478.

This approach has been further explored by its authors resulting in optimized SIMT and partitioned vectorized algorithm CUDASW++ 2.0 [19] with an astonishing performance of up to 17 GCUPS on a GeForce GTX 280 and 30 GCUPS on a dual-GPU GeForce GTX 295.

Likewise, CUDASW++ 3.0 [21] couples CPU and GPU SIMD instructions and carries out concurrent CPU and GPU computations. It employs SSE-based vector execution units as accelerators and employs CUDA PTX SIMD video instructions to gain more data parallelism beyond the SIMT execution model. Evaluation shows that CUDASW++ 3.0 gains a performance improvement over CUDASW++ 2.0 up to 2.9 and 3.2, with a maximum performance of 119.0 and 185.6 GCUPS, on a single-GPU GeForce GTX 680 and a dual-GPU GeForce GTX 690 graphics card, respectively. It also has demonstrated significant speedups over SWIPE and BLAST+. However, the longest query sequence was of length 5,478 to search against the Swiss-Prot protein databases that have the largest sequence length 35,213.

However, most of GPU implementations cannot align sequences longer than 59,000 residues. This is due to the intrinsic SIMD characteristics of the GPG, where pipelining allows a great speedup factor, yet intense memory usage may lead to bottlenecks. This leads to the deployment of other architectures such as many-cores [23]. MC64-ClustalWP2 has been developed recently as a new implementation of the ClustalW algorithm, to align long sequences in architectures with many cores. It runs multiple alignments 18 times faster than the original ClustalW algorithm and can align sequences that are relatively long (more than 10 kb).

Authors proposed a vectorized distance matrix computation algorithm called* DistVect* [8]. The algorithm addresses the problem of building a parallel tool for multicores that produces the alignment of multiple sequences in a short time without using much storage space. The main contribution was the vectorization of all used matrices in computation. Experimentally, the proposed method achieved good ability of aligning large number of sequences through powerful improved storage handling capabilities with efficient improvement of the overall processing time.

## 3. *DistVect* Algorithm

*DistVect* [8] is an accelerated algorithm that computes the distance matrix for aligning huge datasets. It has the advantage of exhausting less space. It takes, as input *N*, sequences {*S*_1_, *S*_2_,…, *S*_*N*_} of average length *L*, with their substitution matrix sbt and the gab cost *g*. It outputs a distance vector, DV, containing the similarity score (distance) for each of the two sequences. It works on vectorizing matrices presented by Liu et al. in [22] and used by Wirawan et al. in [18]. It parallelizes the computations of resolving vectors, taking in account the advantage of the independence of the elements of the minor diagonals of the matrices.

To compute the number of exact matches and make it suitable for a fine-grained parallel implementation, Liu et al. [22] formulated a recurrence relation for the number of exact match computations that is more suitable for implementation using a linear gap penalty. This formula facilitates the calculations without computation of the actual alignment. Given two sequences *S*_*i*_ and *S*_*j*_ of lengths *L*_*i*_ and *L*_*j*_, the distances are computed using the matrices *N*, *H* as shown below:

(1) DM(Si,Sj)=1−N(imax⁡,jmax⁡)min⁡(Li,Lj),N(x,y)={0if  H(x,y)=0,N(x−1,y−1) +m(x,y)if  H(x,y)=H(x−1,y−1)     +sbt(Si(x),Sj(y)),N(x,y−1)if  H(x,y)=H(x,y−1)+g,N(x−1,y)if  H(x,y)=H(x−1,y)+g,H(x,y)=max⁡{0,H(x−1,y−1)+sbt(Si(x),Sj(y)),H(x−1,y)+g,H(x,y−1)+g,m(x,y)={1Si(x)=Sj(y),0otherwise,

where *H*(*x*, 0) = *H*(0, *y*) = *N*(*x*, 0) = *N*(0, *y*) = 0 and 1 ≤ *x* ≤ *L*_*i*_, 1 ≤ *y* ≤ *L*_*j*_.

In [8], the authors proved mathematically that the above equations can be transformed to the following equations using vectors only:

(2) DV(h)=1−kmax⁡min⁡(Li,Lj),Nvx(k)={0if  Vx(k)=0N1x(k−1) +m(k)if  Vx(k)=V1x(k−1)    +sbt(Si(k),Sj(x−k+1))N2x(k)if  Vx(k)=V2x(k)+gN2x(k−1)if  Vx(k)=V2x(k−1)+gVx(k)=max⁡{0,V1x(k−1)+sbt(Si(k),Sj(x−k+1)),V2x(k−1)+g,V2x(k)+gm(k)={1Si(k)=Sj(x−k+1)0otherwise,

where *k*_max⁡_ is the highest score of *N*, which indicates the number of exact matches in the optimal local alignment, 1 ≤ *h* ≤ ⌈*N*(*N* − 1)/2⌉, max⁡(2, *x* − *L*) ≤ *k* ≤ min⁡((*x* + 1), (*L* + 1)), 1 ≤ *x* ≤ (2*L* − 1), *L* is average lengths, and *N*_1_, *N*_2_ are the exact match values in each iteration.

**Figure 1:**
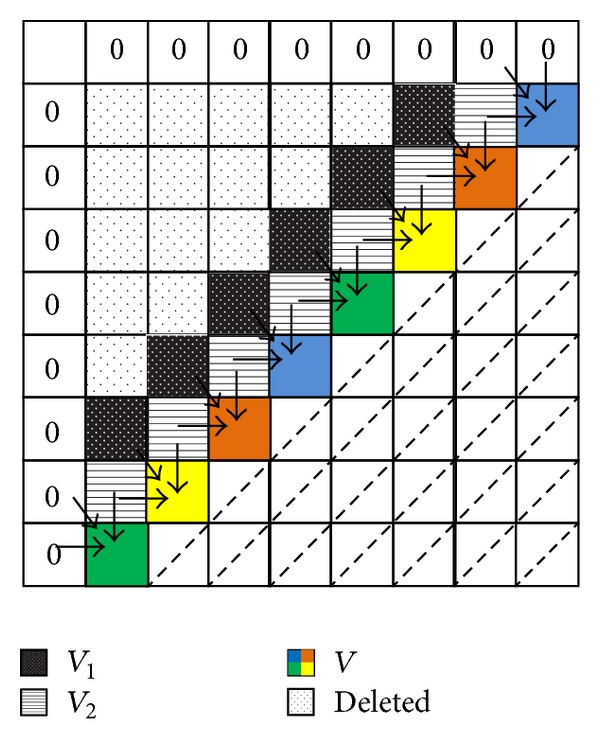
*DistVect* alignment using fine-grain approach.

The main idea was based on the fact that any element in an antidiagonal of *H* needs only the values of three elements from the previous two antidiagonals. This leads to the fact that only three vectors could be sufficient. One vector, *V*, is used as a current vector and the previously calculated vectors, *V*_1_ and *V*_2_, as buffers. Also three vectors, *N*_*v*_, *N*_1_, and *N*_2_, substitute matrix *N*. Finally, the distance matrix, DM, is replaced by a distance vector DV. Each DV element is evaluated in terms of the highest score, *k*_max⁡_.  Figure 1 shows* DistVect* methodology when aligning two sample sequences of length 8.

## 4. Proposed Algorithm

Although* DistVect* implementation on a multicore using OpenMp and multithreading has achieved good speedup compared to ClustalW-MPI [17], it shows load unbalancing. To overcome this problem, we propose using a better computation partitioning technique and accompanying extensions to operate on recent available clusters system that offers more power to parallelism. This is done in order to enhance the performance. In this section, an improved version of* DistVect* is proposed.

*DistVect* computes the similarity scores using only three vectors instead of the matrix DM using fine-grained parallelism and multithreading. It distributes all cells' computations of the antidiagonal vector, *V*, over the available *P* cores. The value of each cell is evaluated in terms of its diagonal neighbor stored at *V*_1_, with its left and upper neighbors stored at *V*_2_, and the maximum value is selected indicating the highest score (see Figure 1).

When analyzing the data dependencies between DM computations, it appears that it may be decomposed through three distinct approaches.

- First, the fine-grain approach, like multithreaded ClustalW [16], is the one in which the computations of each similarity matrix are decomposed. The implementation of this approach in our work [8] shows high sensitivity to the length of sequences and an imbalanced workload among CPU cores.
- Second, the coarse-grain approach partitions DM into blocks (tiles) and assigns the computation of each one to a node, as in [17, 24]. However, this approach is good only when the number of sequences is too large compared to their length and vice versa.
- Third, the hybrid approach (which is used in the proposed algorithm) combines the two previous approaches to benefit from the strong points of both of them.

As mentioned above,* DistVect* algorithm faces some performance challenges when applying the fine-grain approach. The reason is that the size of the vector, *V*, varies during the computation, and the number of cells to be computed at each iteration step is not the same. Thus, when we distribute the cells on the diagonal to *P* cores, we notice that the workload is not well balanced among cores. That is because the number of cells is not always divisible by *P*. Therefore, at each step, some cores have idle time, leading to work overhead on other cores. Consider Figure 1; at iteration 1 (|*V* | = 1) only core 1 works; at iteration 2 (|*V* | = 2) only cores 1 and 2 work. While at iterations 6 and 9 (|*V* | = 6) cores 1 and 2 compute two cells, cores 3 and 4 compute one cell only. Therefore, not all cores compute the same number of cells.

On the other hand, we think this computation model requires a large number of cores for aligning real biological data. That is because our main contribution is based on the acceleration of similarity score computation which is a function of the sequences' length *L*. And we concentrate on dealing with long sequences, but each core calculates one element of *V* at a time. Thus, as the number of cores increases, the parallelization factor will increase, leading to a higher acceleration.

**Figure 2:**
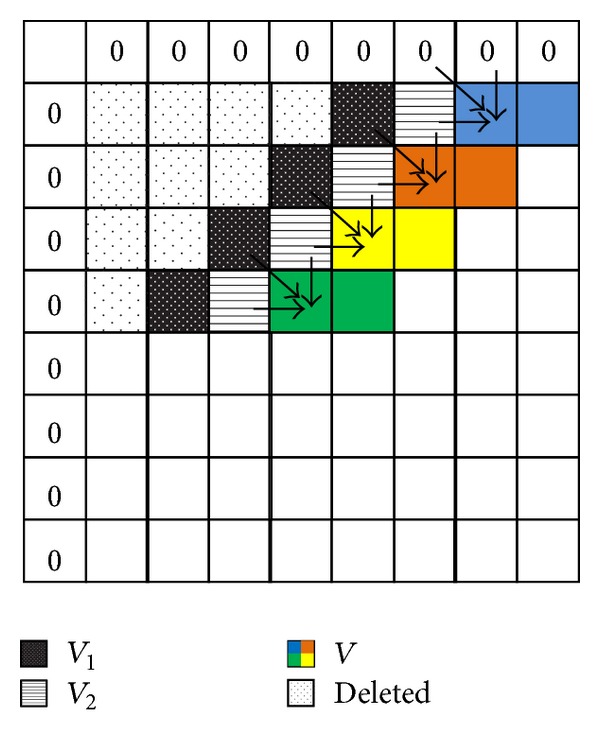
Distance computation using cyclic partitioning.

Figure 2 demonstrates our proposed cyclic partitioning technique, when handling the two sequences considered at Figure 1 with 4 cores. The four cores act in parallel on each of the first four consecutive rows; then they sequentially act on the second consequent 4 rows.

**Figure 3:**
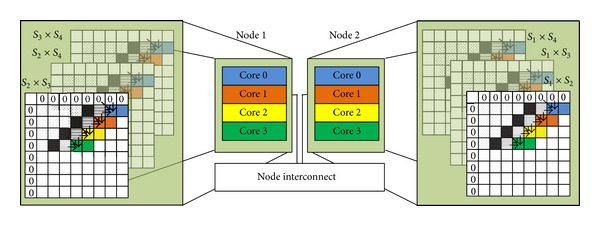
Proposed scheduling on multicore cluster.

Furthermore, all ⌈*N*(*N* − 1)/2⌉ distance computations are partitioned into *M* splits and distributed over the *M* nodes in the cluster system. All *M* nodes work in parallel. Each node will operate sequentially on aligning the set of ⌈*N*(*N* − 1)/2*M*⌉ pairs at its own split, whose size is *n*_split_.  Figure 3 explains how the alignments of 4 sequences of length 8 are scheduled over a cluster system of 2 nodes; each node has 4 cores. In this case, there are 2 splits; each split is of size 3.

The pseudocode of this proposed method is depicted in Algorithm 1. And the distance vector DV is computed using the following modified recurrences:

(3) Vx(k)=max⁡{0,V1x(k−1)+sbt(Si(k),Sj(x−k+1)),V2x(k−1)+g,V2x(k)+g,

(4) Nvx(k)={0if  Vx(k)=0N1x(k−1)+m(k)if  Vx(k)  =V1x(k−1)  +sbt(Si(k),Sj(x−k+1))N2x(k)if  Vx(k)=V2x(k)+gN2x(k−1)if  Vx(k)=V2x(k−1)+g,

where 1 ≤ *k* ≤ *P*.

## 5. Performance Evaluation

The proposed algorithm* DistVect1* has been implemented in C++, using OpenMP and MPI libraries. The introduced hybrid paradigm matches the characteristics of a cluster of multicore system very well. The resulting program has the advantage of coarse-grained parallelism on process level, in which each MPI process is executed on one multicore node. Also, it has the advantage of fine-grained parallelism on a loop level, in which each MPI process spawns a team of threads to occupy the multicore processors when encountering parallel sections of code using OpenMP. However, the obtained results should be carefully analyzed in order to realize the set of items that interfere with the established comparison model.

### 5.1. Experimental Setup

The presented program has been implemented on Sun Microsystems cluster, provided by LinkSCEEM-2 systems at Bibliotheca Alexandrina, Egypt.

This platform specification is as follows:

- 130 nodes and 64 GB memory (the allowed number of nodes for one user is 32 nodes): each node contains two Intel quad core Xeon 2.83 GHz processors (64-bit technology),
- 8 Gbyte RAM, 80 Gbyte hard disk, and a dual port in fin band (10 Gbps),
- a Giga Ethernet Network port: the operating system is 64-bit Linux.

Real protein sequences of different lengths were used for computation. They are available online at NCBI [25]. They comprise a subset of variant family of viruses. Each used benchmark dataset with the average sequence length and the standard deviation is given in Table 1. The standard deviation gives us a numerical measure of the scatter of a dataset. This measure is useful for making comparisons between datasets that go beyond simple visual impressions.

Comprehensive experiments have been conducted on the specified platform using different groups of sequences selected from the indicated datasets. They evaluated the implementation of* DistVect1* versus that of* DistVect* and two popular efficient programs:

- ClustalW-MPI available at http://www.mybiosoftware.com/alignment/3052,
- SSE available at https://sourceforge.net/projects/distmatcomp/.

For SSE2 program, we have used the SSE approach within each MPI process after distributing the overall workload using the proposed scheduling technique, as clarified at Figure 3. Thus, each node applies its multithreading approach on its assigned matrices, one at a time. Bibliotheca Alexandria used platform support Advanced Vector Extensions (AVX), which is used during implementing SSE2 code.

### 5.2. Execution Time, Speedup, and Efficiency

In this subsection, three common performance measurements, the execution time, the speedup, and the efficiency, are used. Parallel execution time represents the elapsed time for complete computation of the distance matrix, including all additions, comparisons, and maximum operations. Parallel speedup is evaluated by the ratio between the parallel execution time of the two involved programs. Parallel efficiency is computed by dividing the corresponding parallel speedups by the number of cores. Results for variable number of available nodes with their analysis are presented below.

**Figure 4:**
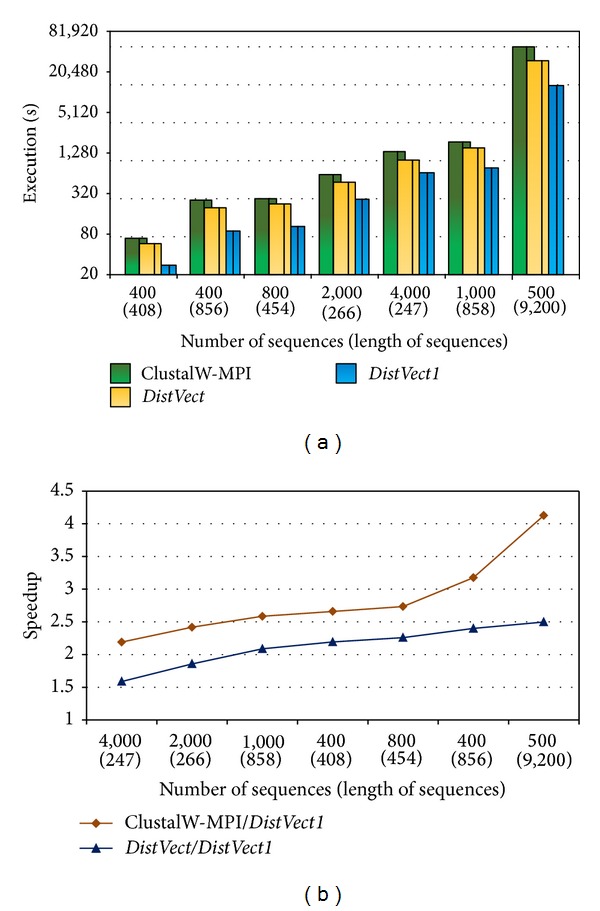
Performance comparisons using one node and 8 cores.

As a start, experiments were executed on a single node with 8 cores. Since the amount of computations required for a sequence depends on its length, multiple lengths of sequences from HIV, Coronaviridae, and HA haemagglutinin were tested. The execution time (in seconds) of the proposed* DistVect1* implementation against ClustalW-MPI and* DistVect* was recorded.  Figure 4(a) shows how the performance of* N(L)* is affected by the sequences' number, *N*, and length, *L*.* DistVect1* shows slightly higher performance than that of the two other programs when *L* ranges in the hundreds and *N* ranges in the thousands. Further, it presented superior performance when *N* is small compared to *L*, and *L* exceeds a thousand. As shown in Figure 4(b), the maximum achieved speedups when comparing set 500(9,200) were (4.1) and (2.5).

**Figure 5:**
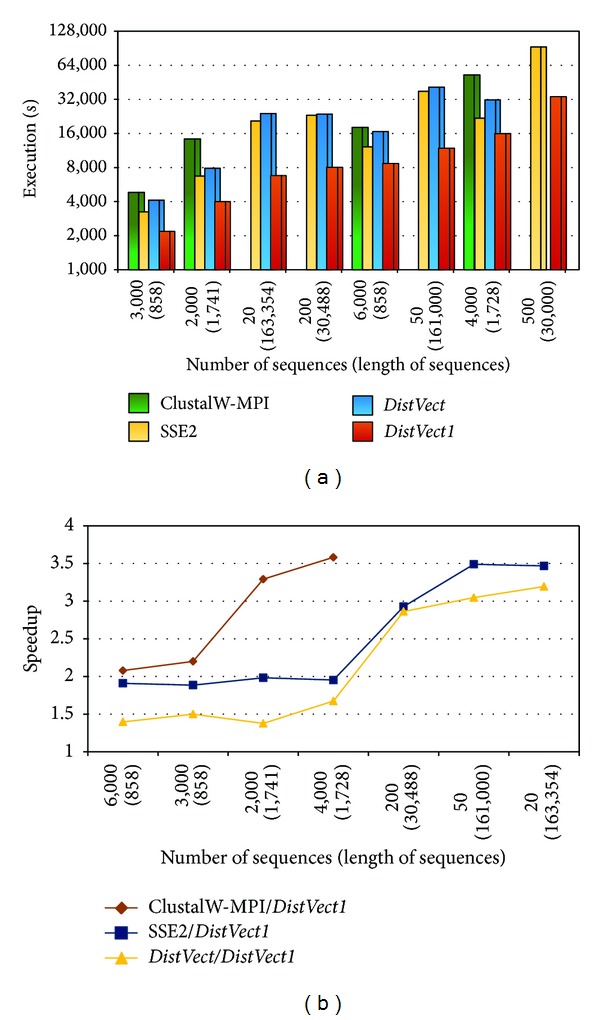
Performance comparisons using 8 nodes.

Next, variant subsets of the specified dataset with different sizes were tested on 8 nodes.  Figure 5 shows that ClustalW-MPI could not handle long sequences with *L* overriding thirty thousand. That is because ClustalW-MPI approach strongly depends on the number of sequences and focuses on the distribution of sequences across nodes without regarding their length. Also,* DistVect* and SSE2 concentrate on the thread level parallelism approach which is based on *L*. As a result, our program performed better than the three programs in all cases and operated smoothly with very long sequences. This accomplishment is due to the perfect vectorization and hybrid partitioning approaches. For example, for comparing set 200(30,488), ClustalW-MPI did not work,* DistVect1* consumed 23,467 sec., SSE2 exhausted 22,949 sec., and* DistVect1* achieved it in 8,017 sec.

**Figure 6:**
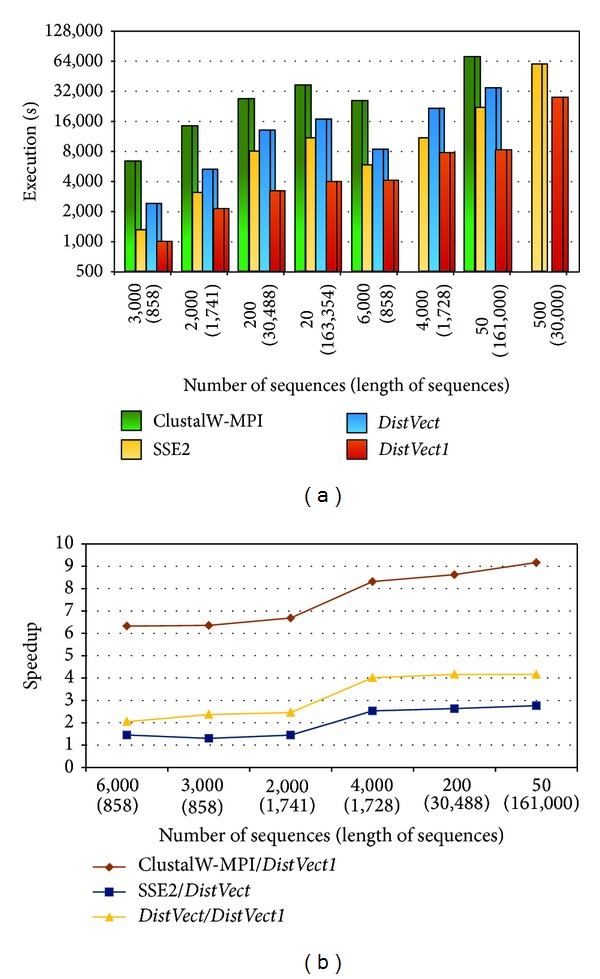
Performance comparisons using 16 nodes.

Also, the same dataset was tested on 16 nodes. Results given in Figure 6 demonstrate that as the number of nodes increases, the performance upgrades. For example, compared to the same set, 200(30,488), ClustalW-MPI works but slowly takes 26,824 sec.;* DistVect* consumed better time, 12,955 sec.; SSE2 exhausted less period, 8166 sec.; and* DistVect1* presents the fastest achievement, 3,226 sec.

**Figure 7:**
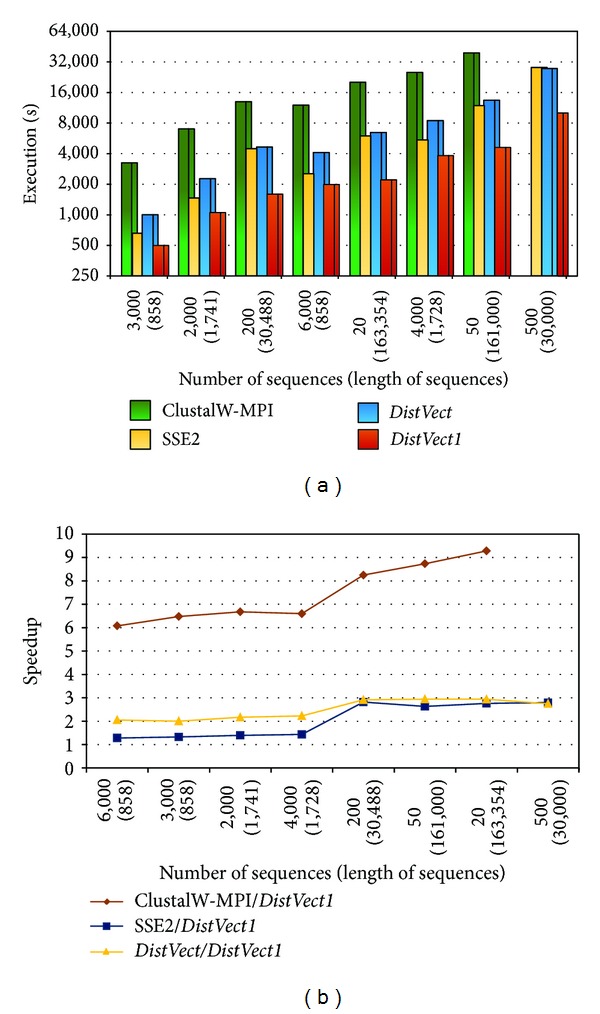
Performance comparisons using 32 nodes.

Finally, when tests were performed on 32 nodes, as seen in Figure 7,* DistVect1's* overall performance is typically much better when compared to other programs. In fact, it exhibits its superiority when *L* is in tens of thousands. The maximum speedup for set 20(163,354) was up to 9, 3, and 2 with respect to the ClustalW-MPI, SSE2, and* DistVect*, respectively.

**Figure 8:**
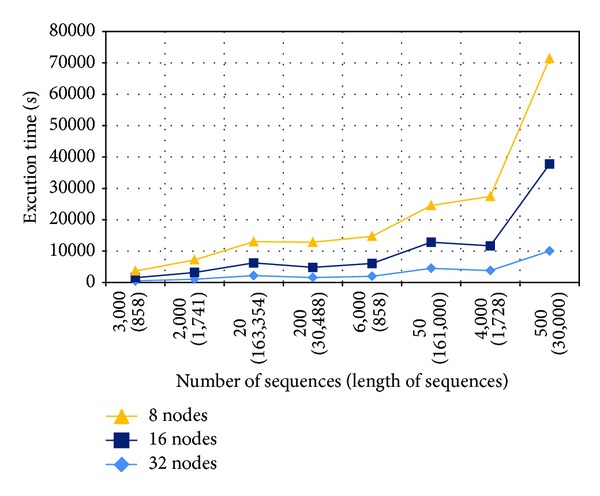
*DistVect1* performance against number of nodes.

To ensure that* DistVect1* execution time decreases as the number of nodes increases, Figure 8 illustrates the relation between the execution time and the number of nodes. As a consequence, it is concluded that* DistVect1* implementation acts almost monotonically, increasing whenever the number of available nodes increases. Therefore, we can state that* DistVect1* execution time is inversely in proportion to the number of nodes.

To emphasize that the proposed* DistVect1* program is more efficient than other implemented programs, the parallel efficiency in terms of the maximum number of available cores has been evaluated. Results are recorded in Table 2. It is clear that* DistVect1* efficiency is monotonically increasing as the length of sequences increases. Thus, we can state that* DistVect1* efficiency is directly in proportion to the sequences' length. In addition,* DistVect1's* supreme efficiency was up to 0.29, 0.086, and 0.092 with respect to the ClustalW-MPI, SSE2, and* DistVect*, respectively, for the longest sequence length (163 k).

### 5.3. GCUPS

A performance measurement commonly used in computational biology is billion cell updates per second (GCUPS). A GCUPS represents the time for a complete computation of one entry in the similarity matrix, including all comparisons additions and maximum operations. We have scanned the datasets with their average length as mentioned above. Our implementation on a cluster of multicores allows handling sequences up to a length of 163 k.  Table 3 compares the corresponding GCUPS performance values.

### 5.4. Space Reduction

The huge growth of sequence databases that exceed current programs' capability and computer systems capacity gives rise to the importance of considering space complexity as an intrinsic performance metric. In the following, we study the exhausted storage space as a function of *L*, the sequence length, and *N*, the number of sequences.* DistVect1* has reduced the overall consumed space by other programs in two ways.

- The compensation of the distance matrix, DM, by the distance vector, DV, reduces space from *N* ^2^ into *h* = *N*(*N* − 1)/2, that is, almost to the half, as shown in Table 4.
- The substitution of matrices *H* and *N* by vector* V's* and three *N*_*v*_′*s* reduces space from 2 × (*L* + 1)^2^ to 6*L*, as shown in Table 5.

### 5.5. Load Balancing and Occupancy

**Figure 9:**
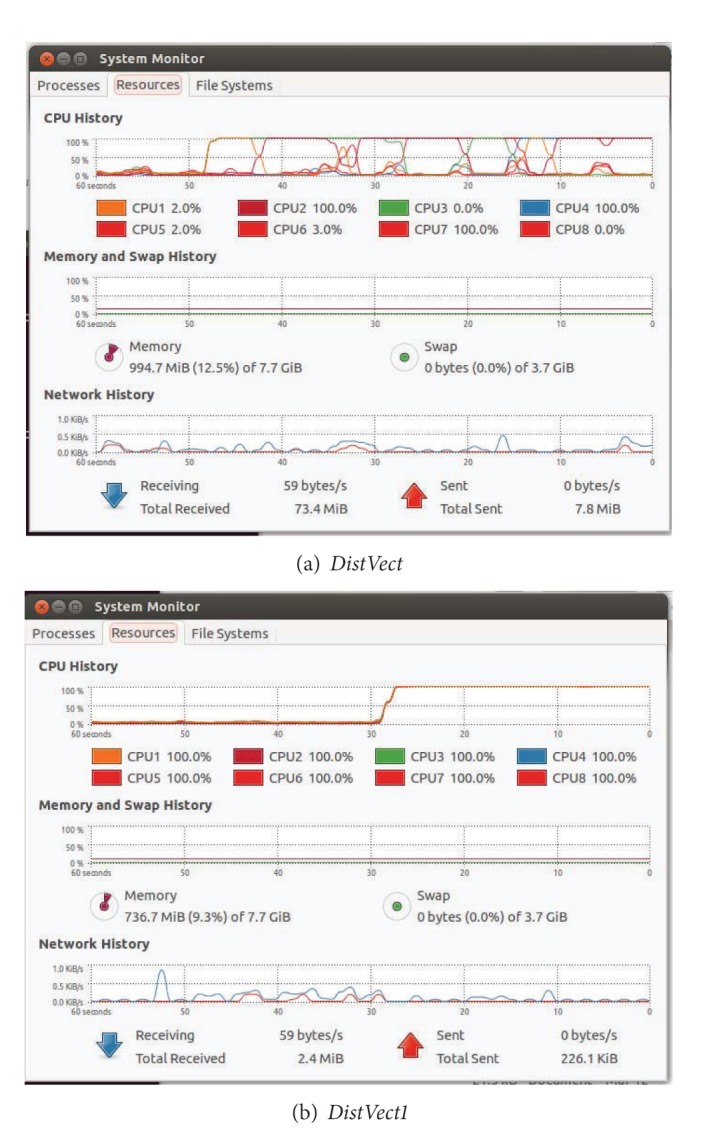
CPUs, memory, and network occupancy.

To study the occupancy of processors and memory, real snapshots have been taken as seen in Figure 9. They record the history of CPUs, memory, and network during execution. They demonstrate how different proposed versions of the algorithm improve load balancing and memory bandwidth. The load unbalancing of* DistVect* is apparent in Figure 9(a). On the contrary, the optimal load balancing of* DistVect1*, with the full use of all CPUs by 100%, is obvious. Also, the reduction of memory occupancy is very clear in Figure 9(b).

As a conclusion, a comparison of* DistVect1* proposed method with studied GPU and cluster implementations discussed in this paper is illustrated in Table 6. For each program, it specifies its platform and records the maximum sequence length that may be handled, the highest speedup, and GCUPS which can be reached. Records certify our presumed idea that although GPU programs are very fast with high GCUPS, they cannot align long sequences. It also proves that our proposal is superlative in handling long sequences with good speedup.

The above presented results reveal the best performance of the* DistVect1* against other implementations. This achievement is due to two main reasons: (1) the superiority of* DistVect1* in using vectors instead of matrices, leading to a better caching, and (2) the optimal use of multicore cluster system because of the proposed partitioning and scheduling techniques, especially when the length of sequences increases, thus obtaining a higher parallelization factor.

## 6. Conclusion and Future Work

In this paper, we presented and evaluated* DistVect1* algorithm, an improved version of the vectorized parallel algorithm* DistVect*, for efficiently computing the distance matrix using multicore cluster systems. The new suggested partitioning and scheduling approaches, integrated with MPI and OpenMP primitives, have achieved a significant improvement to the overall performance. Implementations were conducted on Bibliotheca Alexandria cluster system using 32 nodes (8 cores).

The resulting program,* DistVect1,* was able to compute distances between very long sequences, up to 163 k. It outperforms SSE2 with 3-fold speedup and ClustalW-MPI 0.13 with 9-fold speedup. Its efficiency reaches 0.29, 0.086, and 0.092 over the ClustalW-MPI, SSE2, and* DistVect*, respectively. Moreover, it accomplishes 100% of CPUs occupancy with optimal load balancing and less memory exhaustion. The performance figures also vary from a low of 6.27 GCUPS to a high of 11.69 GCUPS as the lengths of the query sequences increase from 1,750 to 30,500. We believe that, if more cores are provided, a better performance will be achieved and a higher speedup with improved efficiency will be accomplished.

For future work, it is planned to develop an efficient multiple sequence alignment tool as an extension to the proposed work. Furthermore, it is expected to exploit the proposed parallel program for providing better solutions for a class of widely encountered problems in bioinformatics and image processing.

## Figures and Tables

**Algorithm 1:**
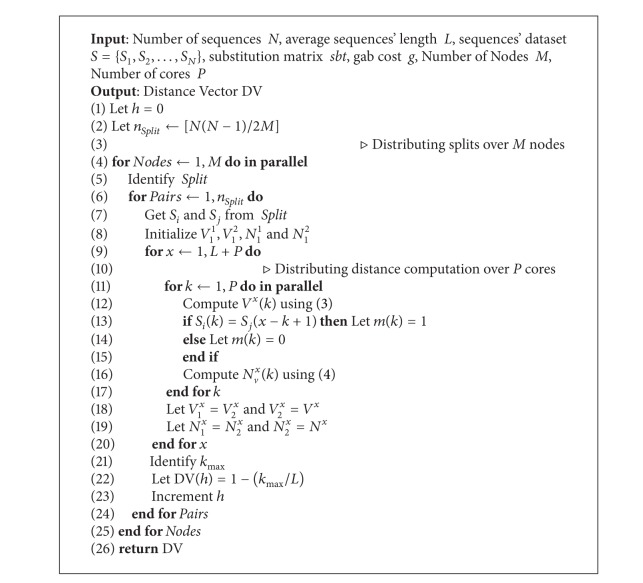
*DistVect1*: Hybrid *DistVect* Algorithm.

**Table 1:** Used benchmark dataset specifications.

Benchmark	Number of sequences	Average length	Standard deviation
Human immunodeficiency virus (HIV)	3,000	858	10.745
	6,000	858	14.632

HA hemagglutinin (influenza B virus)	2,000	1,741	32.604
	4,000	1,728	36.661

Several viruses within the Coronaviridae family	200	30,488	908.534
	500	29,414	1,066.417

Herpesviridae (large family of DNA viruses)	20	163,654	47,330.32
	50	161,000	42,899.81

**Table 2:** Efficiency comparisons using 32 nodes.

Number of sequences (Length of sequence)	ClustalW-MPI	SSE2	*DistVect *
6,000 (858)	0.1899	0.0401	0.0642
3,000 (858)	0.2025	0.0414	0.0625
2,000 (1,750)	0.2087	0.0436	0.0677
4,000 (1,750)	0.2061	0.0449	0.0696
200 (30,500)	0.2578	0.0881	0.0910
50 (161,000)	0.2730	0.0823	0.0919
20 (163,650)	0.2901	0.0863	0.0919

**Table 3:** Performance comparison (in GCUPS) for scanning the datasets.

Number of sequences (Length of sequence)	* DistVect1 *	* DistVect *	SSE2	ClustalW-MPI
20 (163,650)	2.32	0.79	0.84	0.25
2,000 (1,750)	5.82	2.68	4.17	0.87
4,000 (1,750)	6.27	2.81	4.36	0.95
3,000 (858)	6.66	3.33	5.03	1.03
6,000 (858)	6.69	3.26	5.22	1.10
200 (30,500)	11.69	4.01	4.15	1.42
50 (161,000)	7.03	2.39	2.67	0.80
500 (30,000)	10.75	3.92	3.85	0.00

**Table 4:** Storage Comparisons between DV, DM (in MW).

N	20	50	200	500	2,000	3,000	4,000	6,000
DM	0.0004	0.0025	0.04	0.25	4	9	16	36
DV	0.00019	0.001225	0.0199	0.12475	1.999	4.49	7.99	17.998

**Table 5:** Storage comparisons between *H*'s, *V*'s (in MW).

* L *	858	1,728	29,414	161,000
* H*'s +* N*'s	1.475	5.97	173.48	518
* V*'s + *N*_*v*_'s	0.005	0.01	0.176	0.966

**Table 6:** *DistVect1* comparison against other programs.

Software	Platform	Maximum sequence length	Highest GCUPS	Highest speedup
CUDASW++ 2	Dual-GPU GeForce GTX 295	59 k	28 (144–5478)	1.78 with (CUDASW++ 1.0)
CUDASW++ 3	Dual-GPU GeForce GTX 690	59 k	185.60 (144–5478)	3.2 with (CUDASW++ 2.0)
MC64-ClustalWP2	Intel Xeon Quad Core	300 k	0.92	7.76 with (ClustalW-MPI)
SSE2	Cell/BE	0.858 k	0.0058	76.20 with ClustalW sequential
ClustalW-MPI	PC cluster	1100	0.07	14.5 with ClustalW sequential
*DistVect1*	Cluster of multicores	163 k	11.69	9.2 with (ClustalW-MPI)
